# Tissue Biomarkers in Prognostication of Serous Ovarian Cancer following Neoadjuvant Chemotherapy

**DOI:** 10.1155/2014/401245

**Published:** 2014-04-17

**Authors:** Binny Khandakar, Sandeep R. Mathur, Lalit Kumar, Sunesh Kumar, Siddhartha Datta Gupta, Venkateswaran K. Iyer, M. Kalaivani

**Affiliations:** ^1^Department of Pathology, All India Institute of Medical Sciences, Ansari Nagar, New Delhi 110029, India; ^2^Department of Medical Oncology, Dr. B.R. Ambedkar Institute-Rotary Cancer Hospital, All India Institute of Medical Sciences, Ansari Nagar, New Delhi 110029, India; ^3^Department of Gynecology and Obstetrics, All India Institute of Medical Sciences, Ansari Nagar, New Delhi 110029, India; ^4^Department of Biostatistics, All India Institute of Medical Sciences, Ansari Nagar, New Delhi 110029, India

## Abstract

Serous ovarian cancer (SOC) is a significant cause of morbidity and mortality in females with poor prognosis because of advanced stage at presentation. Recently, neoadjuvant chemotherapy (NACT) is being used for management of advanced SOC, but role of tissue biomarkers in prognostication following NACT is not well established. The study was conducted on advanced stage SOC patients (*n* = 100) that were treated either conventionally (*n* = 50) or with NACT (*n* = 50), followed by surgery. In order to evaluate the expression of tissue biomarkers (p53, MIB1, estrogen and progesterone receptors, Her-2/neu, E-cadherin, and Bcl2), immunohistochemistry and semiquantitative scoring were done following morphological examination. Following NACT, significant differences in tumor histomorphology were observed as compared to the native neoplasms. MIB 1 was significantly lower in cases treated with NACT and survival outcome was significantly better in cases with low MIB 1. ER expression was associated with poor overall survival. No other marker displayed any significant difference in expression or correlation with survival between the two groups. Immunophenotype of SOC does not differ significantly in samples from cases treated with NACT, compared to upfront surgically treated cases. The proliferating capacity of the residual tumor cells is less, depicted by low mean MIB1 LI. MIB 1 and ER inversely correlate with survival.

## 1. Introduction

Ovarian cancer is the second most common gynecological cancer worldwide and one of the leading causes of death due to malignancies in females [1]. Incidence of ovarian cancer in India is lower than the western countries and affects postmenopausal females in their sixties [2, 3]. Almost 90% of malignant ovarian tumors arise from the surface epithelium, serous carcinoma being the commonest histological subtype [4–7]. Clinical symptoms are nonspecific and more than 50% of the cases come to attention at an advanced stage with a poor long-term outcome. Conventional treatment of serous ovarian cancer (SOC) comprises surgical removal of tumor, followed by Platinum/Taxane based chemotherapy [8]. Currently, “sandwich therapy,” that is, neoadjuvant chemotherapy (NACT) with interval debulking surgery and postsurgery chemotherapy (CT), is preferred for advanced stage disease (stage IIIC or IV, of the FIGO staging system). The efficacy of this treatment protocol is presently under evaluation [7–9].

A number of prognostic factors for SOCs have been described. The most important ones are FIGO staging and volume of the residual disease after initial cytoreductive surgery [10]. Apart from these, tumor grade, histological subtype, and expression of tissue biomarkers are described in conventionally managed high grade SOC [11–15]. With the growing use of NACT in management of SOCs, it is essential to explore the post-NACT expression of tissue biomarkers and evaluate their utility in prediction of response to therapy and prognosis. Utility of p53, ER, PR, and MIB 1 LI has been reported for this group in one study [9]. The present study evaluates these and other biomarkers like Bcl2, E-cadherin, and Her-2/neu in post-NACT samples which has not been evaluated earlier.

## 2. Materials and Methods

This study was a combined retrospective and prospective study including cases from January 2001 to December 2010 seen in the Departments of Medical Oncology and Pathology, AIIMS, New Delhi. One hundred cases of SOC were included: fifty treated with 3 cycles of NACT followed by surgery and 3 cycles of CT (NACT group) and fifty patients who underwent upfront surgery (US) followed by 6 cycles of CT (US-CT group).

Formalin-fixed paraffin-embedded blocks were prepared from the surgical resection specimens of both groups and Hematoxylin and Eosin (H & E) stained sections were examined. Appropriate blocks with adequate viable tumor tissue were selected for immunohistochemical (IHC) analysis. IHC was performed using commercially available monoclonal antibodies for p53 (Neomarkers clone; RM-9105-S, dilution; 1 : 200), Bcl2 (Neomarkers clone; MS-123-P1, dilution; 1 : 150), ER (Neomarkers clone; MS-750-S, dilution; 1 : 200), PR (Neomarkers clone; MS-390-S, dilution; 1 : 100), E-cadherin (Novocastra clone; NCL-E-CAD, dilution; 1 : 50), Ki-67 (Neomarkers clone; RM-9105-S1, dilution; 1 : 400), and Her-2/neu (Neomarkers clone; MS-441-S, dilution; 1 : 400). Sections were cut from the selected blocks on poly-L-lysine coated slides and deparaffinized and antigen retrieval was done. Overnight incubation with primary antibody at 4°C was performed. Polymer based biotinylated secondary antibody followed by DAB (Di-amino Benzidine) visualization and Hematoxylin counterstain were done. With each batch, appropriate positive and negative controls (omitting the primary antibody) were also run. IHC slides were reviewed and semiquantitative scoring was done by two pathologists (BK and SM). IHC finding of p53, ER, PR, Bcl2, and E-cadherin was interpreted as 0 for no staining, 1+ for staining in up to 30% of cells, 2+ for staining in >30 to ≤60% of cells, and 3+ for staining in >60% of cells. For scoring of Her-2/neu, the interpretation criterion routinely used in carcinoma breast was applied. MIB1 Labeling Index (MIB 1 LI) was calculated by counting 500 cells in the highest proliferating area at 400x magnification.

Survival and follow-up data was retrieved. Months of survival and outcome at the end of follow-up period were noted by means of clinical examination, radiological evaluation, and cytological/biopsy samples.

Statistical analysis was done using Stata 11.0 software. Nonparametric tests (Pearson chi square and Fisher exact test) and Kaplan-Meier analysis for period of survival were applied.

## 3. Results

### 3.1. Age and Stage Distribution

The mean age of the patients was fifty years. Most patients were stage IIIC and only 7 cases were stage IV (3 in NACT group, 4 in US-CT group).

### 3.2. Morphological Analysis

**Figure 1:**
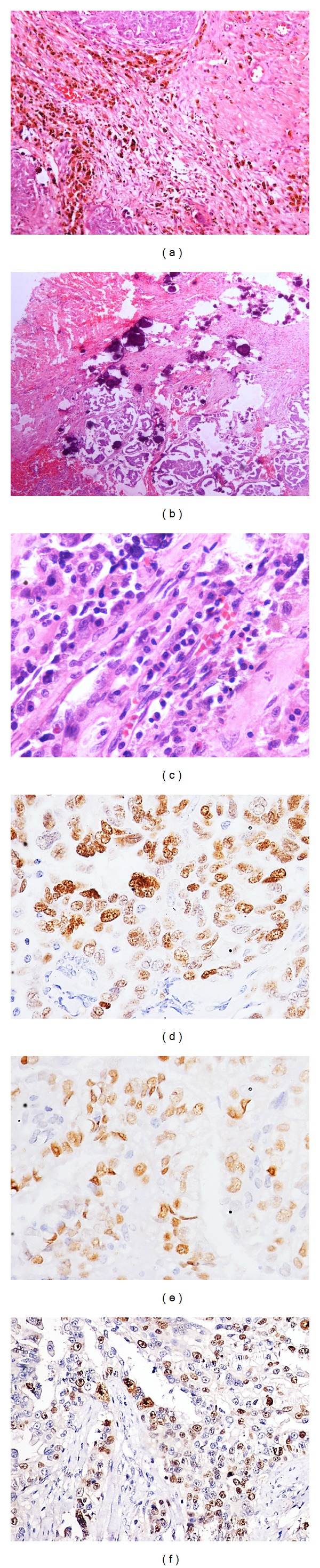
Postneoadjuvant chemotherapy serous ovarian carcinoma showing stromal fibrosis with infiltration of eosinophils, plasma cells (H & E stain; (a) ×100), psammomatous calcification (H & E stain; (b) ×100), and infiltration of plasma cells (H & E stain; (c) ×200). Photomicrograph of postneoadjuvant chemotherapy serous ovarian carcinoma showing immunohistochemical expression (brown nuclear positivity) of p53 ((d) 400x), estrogen receptor ((e) 400x), and MIB 1 ((f) 200x).

A detailed morphological analysis was done on all the tumor samples before performing immunohistochemistry. The tumors receiving NACT showed significantly more stromal fibrosis (Figure 1(a)) with areas of hyalinisation, psammomatous calcification (Figure 1(b)), inflammatory cells (Figure 1(c)), and foamy macrophages. The residual tumor cells in post-NACT samples showed bizarre nuclei with variable degree of cytoplasmic degenerative changes.

### 3.3. Immunohistochemical Analysis

p53 positivity was detected in 60% of the cases (29 in NACT group, 31 in US-CT group; Table 1; Figure 1(d)), and 75% of the positive cases had a diffuse and widespread (2+ and 3+) staining. No difference in the intensity of p53 positivity was found across the two groups (Table 2). Estrogen receptor expression did not differ significantly between the treatment groups (48% in NACT group, 42% in US-CT group; Table 1). ER (Figure 1(e)) was diffusely expressed (2+ and 3+) in 31% cases (*n* = 14 and 17, US-CT and NACT group, resp., Table 2). Progesterone receptor (PR) was expressed in 10% cases (*n* = 6 and 4 in US-CT and NACT group, resp.), with only weak focal positivity (Tables 1 and 2). 52% of the cases were both ER and PR negative (*n* = 28 and 24, US-CT and NACT group, resp., Table 3) and only 7% of the cases were both ER and PR positive (*n* = 5 and 2, US-CT and NACT group, resp., Table 3). MIB 1 LI was significantly higher in the US-CT group (*P* < 0.05, Table 4). Only 9 cases of the NACT group showed a high MIB 1 LI which included 2 cases with MIB 1 LI > 60% and 7 cases with MIB1 LI > 30% (Table 4). Mean MIB 1 LI was 20% and 40% in NACT (Figure 1(f)) and US-CT groups, respectively. Bcl2 expression was observed in 12% of the cases (8 in NACT group and 4 in US-CT group, resp.). 10 cases (*n* = 4 and 6, US-CT and NACT group, resp.) revealed focal positivity (1+) and only 2 cases of NACT group showed 2+ Bcl2 positivity (Table 2). E-cadherin was focally (1+) positive in 27% of the cases without any difference across the treatment categories. Only 2 cases of US-CT group showed 2+ E-cadherin positivity (Table 2). All the tumor samples included in the study were negative for Her-2/neu.

### 3.4. Survival Analysis

**Figure 2:**
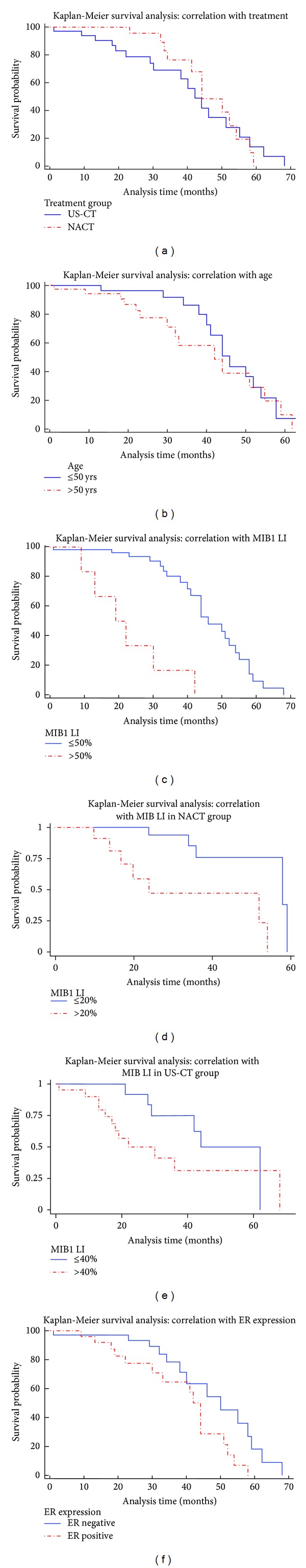
Kaplan-Meier survival analysis curves depicting correlation of survival outcome with treatment (a), age (b), MIB 1 LI (c), mean MIB 1 LI in the neoadjuvant chemotherapy group (d), mean MIB 1 LI in the conventional treatment group (e), and estrogen receptor (f).

Survival analysis was done for cases with available follow-up data in 62 patients. Thirty patients died during the study period, 12 in NACT group and 18 in US-CT group. Median overall survival of patients in the NACT group was 32 months. For the US-CT group the median overall survival was 29 months (Figure 2(a)).

For analyzing the correlation of survival with age, median age (50 years) was taken for comparison. Longer median survival of 46 months was observed in patients ≤50 years of age, in comparison to 42 months in patients >50 years of age (*P* = 0.34; Figure 2(b)). Prognosis was poor in patients with stage IV disease. Survival was better in patients receiving NACT, as compared to the patients who received conventional therapy; however the difference was not statistically significant (*P* = 0.6, Figure 2(a)).

Patients with MIB 1 LI > 50% had median survival of 20.5 months with a significant poor overall survival (*P* < 0.001; Table 5, Figure 2(c)). Further survival analysis was done separately in the two treatment categories taking mean MIB 1 LI for comparison. In both the treatment categories, overall and median survival was longer in cases with a lower MIB 1 LI (Figures 2(d) and 2(e)). Out of all the other biomarkers analyzed, significantly poor overall survival was seen in the cases expressing estrogen receptor (*P* = 0.0316; Table 6, Figure 2(f)).

## 4. Discussion

Ovarian cancer is the second most common cause of gynecological malignancy in developed countries [16, 17]. The five-year survival rate is considerably better in early stages (around 90%) and relatively dismal (10–30%) in advanced stage [18]. The standard protocol for management of advanced stage SOC is upfront surgery (US) followed by CT. In the recent years, “sandwich therapy” is being deployed [7–9, 19] with increasing frequency worldwide. This study was conducted to analyze the expression of tissue biomarkers in post-NACT samples and also to correlate expression pattern of these tissue biomarkers with survival.

### 4.1. Expression of Tissue Biomarkers

Study by Miller et al. (*n* = 18) described no immunophenotypic difference in post-NACT ovarian carcinomas in comparison to that of native neoplasm [9]. They analyzed CK7, CA125, WT1, ER, p53, and p16. In the current study we have analyzed seven tissue markers in a large number of cases (*n* = 100). Apart from the markers which have been evaluated earlier (p53, ER, PR, and MIB 1), this is the first study that describes the expression of Bcl2, E-cadherin, and Her-2/neu in post-NACT samples.

The most frequent molecular alterations in ovarian carcinoma are p53 mutation. Mutations, most commonly missense, are more common in advanced disease [20]. Similar results were also observed in study done by Ferrandina and coworkers [21]. In our study overall 60% cases were p53 positive, with no significant difference in expression pattern between the two groups, findings similar to study done by Miller et al. [9].

The ovarian neoplasms are characterized by changes in their receptor status. They can either be primarily receptor negative or may lose the receptors with disease progression and expression varies with histological subtypes [22–25]. In this study, ER was expressed in 45% of SOCs without any statistically significant difference across the treatment groups. Study by Miller and coworkers showed diffuse ER positivity in 90% cases without any difference in expression in the postneoadjuvant tumor samples [9]. Expression of PR observed in this study was very low, with PR being positive in only 10 cases. In contrast to the extent of ER expression, our cases showed only focal weak expression of PR. In a study done by Lee and his coworkers on 322 ovarian cancers, serous subtype was mostly ER positive (77.3%), while PR was more frequently expressed (64.2%) in endometrioid cancers [14].

Loss or altered expression of E-cadherin is responsible for tumor dedifferentiation and invasiveness, which plays an important role in tumor progression in epithelial tissues [26, 27]. In epithelium tumors of ovary, E-cadherin expression is more frequently reported to occur in the cases without metastasis [28]. E-cadherin expression was low in our series being positive in 29 cases. No statistically significant difference was observed in expression of E-cadherin between the two groups.

Her-2/neu has been found to have significant prognostic and predictive value in breast cancer [29]. Its amplification is also seen in several other tumors and has been correlated with a poor prognosis. The results in ovarian cancer regarding Her-2/neu overexpression show wide variability. Expression of Her-2/neu in ovarian cancer ranging from 8 to 66% has been reported in various studies [30–35]. The reason is attributed to the different detection methods, namely, immunohistochemistry (IHC), fluorescence in situ hybridization (FISH), or chromogenic in situ hybridization (CISH) used. Differences in the sources of tissue material and tumor heterogeneity may also be responsible for the wide variability [36, 37]. In this study Her-2/neu expression has been analyzed by IHC with positive control slides (3+ Her-2/neu positive carcinoma breast) being run with every batch of IHC done on SOC. All our cases were Her-2/neu negative, with only occasional tumor cells showing faint nonspecific cytoplasmic staining.

The Bcl2 gene increases the survival of the cell by inhibiting apoptosis [38]. Study done by Chan and coworkers revealed that Bcl2 is expressed strongly in the surface epithelium of normal ovaries and benign and borderline ovarian tumors but weakly in the malignant tumors [5]. Baekelandt and coworkers demonstrated 39% positivity of Bcl2 in ovarian cancer [39]. In this study Bcl2 positivity was found in 12% of the cases, with 8% cases from US-CT group and 16% from NACT group. None of the cases showed diffuse strong widespread reaction. No difference in the expression pattern of Bcl2 was found between groups.

Ki-67 is a nuclear antigen used as an indicator of proliferation since it is expressed during G1, S, M, and G2 periods of cell cycle, & absent in the G0, quiescent state of the cell. MIB 1 (Mindbomb 1) is the commonly used antibody on formalin fixed paraffin-embedded tissues [40–42]. In our study, a lower MIB 1 LI (<30%) was found in 82% of the patients receiving NACT (mean MIB1LI of 20%). MIB 1 LI was significantly higher in the patients of US-CT group where most of the patients had MIB 1 LI in the range of 31–60%, with a mean of 40%. Study by Miller and coworkers revealed a reduction in MIB 1 LI in cases displaying significant changes in tumor morphology following NACT [9].

### 4.2. Effect of Treatment on Survival

As already mentioned the standard management is upfront surgery followed by postoperative chemotherapy. As an alternate treatment option, some oncologists treat advanced stage cases with NACT before the cytoreductive surgery. Meta-analysis by Bristow (*n* = 835) showed a poorer outcome in patients with NACT in comparison to primary debulking surgery [43]. A randomized trial has been conducted with advanced stage patients (*n* = 718) to compare the outcome of “sandwich therapy” with primary debulking surgery. The study revealed almost similar median survival in both the treatment groups, 29 months in the patients undergoing primary surgery and 30 months in the NACT group [44]. Our results are similar to this study. Various other studies have suggested that survival of patients of advanced ovarian cancer treated with NACT is similar to those undergoing primary surgery. However, improvement in the performance status of the patients with decreased operative morbidity has been observed. It has also been observed that the number of patients attaining optimal cytoreduction is increased with the institution of NACT [45–48]. Further studies are required to document exact stand of NACT in the treatment of advanced stage ovarian cancer.

### 4.3. Correlation of Survival with Tissue Biomarkers

Tissue biomarkers can assist in planning treatment and also help in predicting long-term outcome. The importance of tissue biomarkers in predicting prognosis of ovarian cancer in conventionally treated cases has been well documented. However their role in predicting prognosis following NACT has not yet been described in literature. This is the first study exploring the significance of seven tissue biomarkers. We found two biomarkers with statistically significant results, MIB 1 and ER.

Higher expression of Ki 67 antigen significantly correlated with poor survival in our study. We found that patients with a MIB 1 LI > 50% had poorer overall survival. Further we analysed correlation of MIB 1 LI in both the treatment groups separately. We found significantly poorer survival in the NACT group when MIB 1 LI was > 20%. Garzetti and coworkers found a higher level of Ki 67 antigen expression in cystadenocarcinoma, in comparison to benign and borderline tumors. Ki 67 expression negatively correlated with survival and a poor disease free survival was observed in cases with higher MIB 1 LI [49]. Study by Kaern and coworkers on advanced stage ovarian cancer patients (*n* = 51) revealed overexpression of Ki-67 was associated with bad prognosis [50].

In our study overall survival was better in estrogen receptor negative cases (*P* = 0.03). Expression of PR has been associated with better prognosis [14]. However, no consensus on prognostic significance of steroid hormone receptor expression in ovarian tumors has been reached yet. ER and PR expression do not significantly change with neoadjuvant chemotherapy and their expression may vary with disease progression along with some difference in survival. More studies are required to conclusively determine the exact role of these two steroidal hormones.

No statistically significant difference in survival was found in our study between p53 negative and positive patients in either of the groups. Many studies have shown that alteration of p53 (mutation/overexpression) status in ovarian cancer does not have a consistent relationship with response to therapy/survival [51–53]. Bartel and coworkers demonstrated patients with normal p53 have a longer survival time compared to patients expressing mutated p53 [54]. The absence of a clear association of p53 alterations with patient outcome may reflect the underlying complexities, and mutations may represent only a subset of the functional p53 alterations.

A reduced expression of E-cadherin has been correlated with a higher tumor grade, presence of peritoneal seeding, and low overall survival rate [55]. Study by Dian et al. on ovarian carcinoma samples showed significant association of E-cadherin expression with grading and FIGO surgical staging (FIGO I + II versus FIGO III + IV, *P* = 0.020). They found strong E-cadherin expression was less in tumors with a higher grade [56]. They also showed that cases with stronger E-cadherin staining intensity had better progression free, cause-specific, and overall survival, though the data was not statistically significant [56]. No statistically significant difference in survival was seen with respect to E-cadherin expression in our study.

In our study we found a longer median survival in cases with Bcl2 positivity, in comparison to Bcl2 negative cases. Similar trend was observed on further analysis among the treatment groups separately. Studies on Bcl2 in relation to prognosis are not uniform. Some studies have failed to demonstrate any significant correlation with survival [57]. On the other hand few studies have shown high levels of Bcl2 expression to be associated with lower chances of response to chemotherapy and a shorter survival [58, 59]. Study done by Kupryjańczyk and coworkers (*n* = 229) revealed a negative correlation of Bcl2 expression with survival of the patients [59]. In contrast to these findings some studies have shown an improved survival in patients with a high level of Bcl2 expression [39]. The expression of Bcl2 and its correlation with survival depends on various factors including host response, level of p 53 expression, tumor grade, and biological behavior of the tumor.

This study analyzes the expression of tissue biomarkers in SOCs across the conventionally treated and the neoadjuvant chemotherapy treated group of patients. Tumor cells differ significantly in their proliferation capacity following NACT, indicated by a lower MIB 1 labeling index in post-NACT samples. In the present study, MIB 1 labeling index and ER expression inversely correlated with survival and could be useful in predicting treatment response and prognostication. The expression of most of the other tissue biomarkers in serous ovarian cancer cases treated with NACT did not differ significantly from the cases managed without preoperative chemotherapy. However, more studies are required to validate the role of tissue biomarkers as predictors of survival and prognosis following NACT.

## Figures and Tables

**Table 1:** Comparison of expression of tissue biomarkers across US-CT and NACT groups.

Biomarker	US-CT group (%)	NACT group (%)	*P* value
p53	31 (62%)	29 (58%)	0.419
ER	21 (42%)	24 (48%)	0.344
PR	6 (12%)	4 (8%)	0.37
Bcl2	4 (8%)	8 (16%)	0.178
E-Cadherin	16 (32%)	13 (26%)	0.33
Her-2/neu	0	0	—

**Table 2:** Comparison of biomarkers score across the treatment groups.

Biomarker	Treatment group	Scoring of biomarker				*P* (US-CT versus CT group)
		0	1	2	3	
p53 (*n* = 100)	US-CT group (*n* = 50)	19	8	10	13	0.95
	NACT group (*n* = 50)	21	7	8	14	

ER (*n* = 100)	US-CT group (*n* = 50)	29	7	8	6	0.238
	NACT group (*n* = 50)	26	7	15	2	

PR (*n* = 100)	US-CT group (*n* = 50)	44	5	1	—	0.741
	NACT group (*n* = 50)	46	4	—	—	

E-Cadherin (*n* = 100)	US-CT group (*n* = 50)	34	14	2	—	0.495
	NACT group (*n* = 50)	37	13	—	—	

Bcl2 (*n* = 100)	US-CT group (*n* = 50)	46	4	—	—	0.304
	NACT group (*n* = 50)	42	6	2	—	

**Table 3:** Comparison of expression of ER/PR.

ER/PR expression	US-CT group (*n* = 50)	NACT group (*n* = 50)
ER−/PR−	28	24
ER−/PR+	1	2
ER+/PR−	16	22
ER+/PR+	5	2

**Table 4:** Comparison of MIB 1 LI*.

MIB LI*	US-CT group (*n* = 50)	NACT group (*n* = 50)	*P* value
≤30%	16 (32%)	41 (82%)	0.001
31–60%	29 (58%)	7 (14%)	
>60%	5 (10%)	2 (4%)	

*LI: labeling index.

**Table 5:** Survival Analysis: comparison with MIB1 LI*.

MIB 1 LI (*n* = 62)	Death (*n* = 30)	Median survival (Months)	*P* value
MIB 1 LI ≤ 50% (*n* = 55)	24	46	0.001
MIB 1 LI > 50% (*n* = 7)	6	20.5	

*LI: labeling index.

**Table 6:** Survival analysis: comparison with ER expression.

ER expression (*n* = 62)	Death (*N* = 30)	Median survival (months)	*P* value
ER positive (*n* = 26)	16	44	0.031
ER negative (*n* = 36)	14	50	
